# Facile Synthesis of Novel V_0.13_Mo_0.87_O_2.935_ Nanowires With High-Rate Supercapacitive Performance

**DOI:** 10.3389/fchem.2019.00595

**Published:** 2019-09-04

**Authors:** Haishun Jiang, Wenjing Sun, Wenyao Li, Zhe Wang, Xiying Zhou, Zexing Wu, Jinbo Bai

**Affiliations:** ^1^School of Material Engineering, Shanghai University of Engineering Science, Shanghai, China; ^2^College of Chemistry and Molecular Engineering, Qingdao University of Science and Technology, Qingdao, China; ^3^Laboratoire Mécanique des Sols, Structures et Matériaux, CNRS UMR 8579, Ecole Centrale Supelec, Université Paris Saclay, Châtenay-Malabry, France

**Keywords:** molybdenum–vanadium oxides, nanowires, hydrothermal, high rate, supercapacitors

## Abstract

Binary metal oxides composed of molybdenum–vanadium oxides are promising candidates for supercapacitors. Here, we report the synthesis of one-dimensional V_0.13_Mo_0.87_O_2.935_ nanowires through a facile one-step hydrothermal method. This nanowire presented a high specific capacitance of 394.6 F g^−1^ (1 mV s^−1^) as an electrode applied to the supercapacitor. Importantly, this electrode showed a perfect rate capability of 91.5% (2 to 10 A g^−1^) and a continuous verified outstanding cyclic voltammetry of 97.6% after 10,000 cycles. These superior electrochemical properties make the synthesized V_0.13_Mo_0.87_O_2.935_ nanowires a prospective candidate for high-performance supercapacitors.

## Introduction

Due to overconsumption of non-renewable resources and the growing threat of global warming, reliable and clean energy supplies, such as the secondary battery and supercapacitor (SC) science and technology, are in urgent need of a breakthrough (Liu et al., 2016; Salanne et al., 2016; Liu M. et al., 2018; Liang et al., 2019). SCs are becoming more appealing than ever because of their rapid recharge capabilities, high power density, and durable life cycles (Salanne et al., 2016; Du et al., 2018; Kirubasankar et al., 2018; Ho and Lin, 2019; Le et al., 2019; Ma et al., 2019; Yang L. et al., 2019). It is well-established that three main electrode materials include conducting polymer, transition metal oxide, and carbon materials (Jabeen et al., 2016a,b; Chen et al., 2017; Li et al., 2018; Idrees et al., 2019). In this regard, transition metal oxides can increase the efficiency and improve the specific capacitances compared to conducting polymers and carbon materials (Yang et al., 2015; Fu et al., 2016; Qin et al., 2016a,b; Meng et al., 2017; An and Cheng, 2018). Unfortunately, it has either insufficient electrochemical stability or low conductivity, which still greatly hampers their widespread applications in SCs (Jiang et al., 2012). Therefore, an innovative material that can be applied as a significant electrode material in the field of SCs is still needed.

In the last few years, binary metal oxides with stoichiometric or even nonstoichiometric composition such as NiCo_2_O_4_ (Ma et al., 2016), NiFe_2_O_4_ (Yu et al., 2014), and MnCo_2_O_4.5_ (Hu et al., 2019) have achieved efficient energy storage. It stems from its defect–effect mechanisms (Ellis et al., 2007; Wang et al., 2017) or possible jump processes (Hu et al., 2012; Li et al., 2018; Yang Y. et al., 2019) that provided the needed efficient electron conductivity. Also, the electrochemical behavior of these binary metal oxides is different to simple metal oxides attributed to their composition, including the species and ratios of elements. In particular, binary metal oxides based on molybdenum oxides or vanadium oxides are also regarded as a potential candidate for SCs. Many binary metals–molybdenum oxides, such as NiMoO_4_ (Cheng et al., 2015), a-MnMoO_4_ (Purushothaman et al., 2012), CoMoO_4_•0.9H_2_O (Liu et al., 2014), and NiMoO_4_ (Mehrez et al., 2019), and binary metal–vanadium oxides, such as β-Na_0.33_V_2_O_5_ (Hong Trang et al., 2014), Li_3_VO_4_ (Iwama et al., 2016), and BiVO_4_ (Patil et al., 2016; Guo et al., 2019), have been prepared for high-performance SCs. Despite the tremendous efforts that have been made on the electrode materials for these binary metal oxides, researchers continue to explore the performance of the electrode material for sustainable, low-cost, and clean energy storage and conversion technologies. Especially, binary metal oxides composed of molybdenum–vanadium oxide are also expected to be of favorable potential as SCs. However, such reports are rare.

Herein, we report a simple preparation of one-dimensional V_0.13_Mo_0.87_O_2.935_ nanowires through a one-step hydrothermal method. This nanowire electrode exhibits a high specific capacitance of 394.6 F g^−1^ (1 mV s^−1^) as an electrode material in SC. Additionally, this electrode showed a rate capability of 91.5% (2 to 10 A g^−1^) and an outstanding cycle stability (97.6% after 10,000 cycles). Therefore, one-dimensional V_0.13_Mo_0.87_O_2.935_ nanowires have been prepared and applied as a high-performance SC electrode material.

## Experimental

### Preparation

Firstly, the molybdenum powder (Mo, 0.192 g, 2 mmol) was mixed with 37 ml of deionized H_2_O and 3 ml of hydrogen peroxide at room temperature and then continuous stirred till the solution became light yellow. After that, 0.088 g of ammonium vanadate (NH_4_VO_3_, 0.75 mmol) was added to the solution until the solid powder was completely dissolved. Then, the resulting solution was decanted into a Teflon reaction kettle and heated in oven at 200°C for 48 h. After cooling to room temperature, the obtained crude products were treated with 2 M nitric acid. Finally, the nanowires were collected through washing with distilled H_2_O till neutral and then dried under air at 60°C for 18 h.

### Material Characterizations

The X-ray diffractometer (XRD; with Cu-Kα radiation) presented the structure and phase of one-dimensional V_0.13_Mo_0.87_O_2.935_ nanowires. The nanowires' morphological feature was studied by a scanning electron microscope (SEM; S-4800) and a transmission electron microscope (TEM; JEM-2100F). Compositions of the samples were tested by X-ray photoelectron spectroscopy (Thermo ESCALAB 250XI). An automated nitrogen adsorption analyzer (ASAP 2020, Micromeritics, America) presented N_2_ adsorption–desorption isotherm under the 77 K conditions.

### Electrochemical Characterizations

Electrochemistry performances were tested in three electrode systems with 1 M Na_2_SO_4_ electrolyte using Autolab potentiostat (PGSTAT302N). A saturated calomel electrode (SCE) was used as the reference electrode and a platinum (Pt) foil was used as the counter electrode. The working electrode was a mixture of one-dimensional V_0.13_Mo_0.87_O_2.935_ nanowires, acetylene black, and polyvinylidene fluoride (PVDF) according to a certain mass ratio (80:15:5) in a few *N*-methyl pyrrolidinone (NMP). After the mixture was stirred for 24 h, the formed slurry was dripped on graphite paper and then vacuum dried at 60°C for 15 h. Cyclic voltammetry (CV) measurement was carried out in a voltage range of 0–1.0 V at different sweeping rates (1, 5, 10, 25, 50, 75, and 100 mV s^−1^), and galvanostatic charge–discharge (GCD) was tested at different current densities (2, 4, 6, 8, and 10 A g^−1^). EIS data are obtained at a frequency from 10^−2^ to 10^5^ Hz with an AC amplitude of 5 mV.

## Results and Discussions

**Figure 1:**
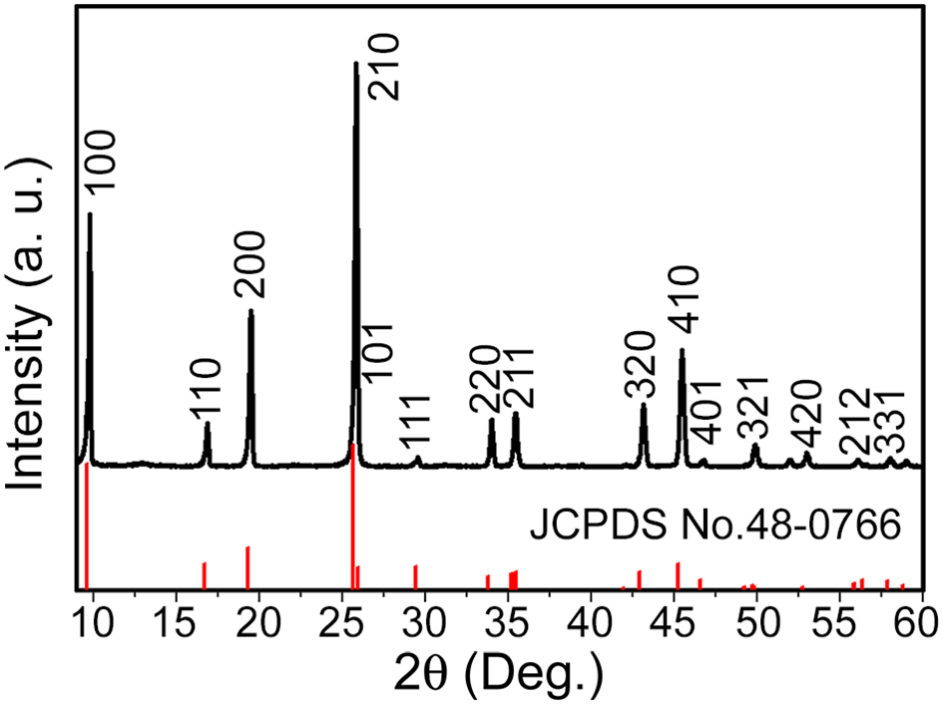
XRD spectrum of the one-dimensional V_0.13_Mo_0.87_O_2.935_ nanowires.

In the present work, the phase for one-dimensional V_0.13_Mo_0.87_O_2.935_ nanowire was first characterized. The XRD spectrum for the prepared product is indicated in Figure 1 in that all diffraction peaks matched a hexagonal phase of one-dimensional V_0.13_Mo_0.87_O_2.935_ nanowires (JCPDS card No. 48-0766). No characteristic peaks from impurity have been detected, suggesting that the pure one-dimensional V_0.13_Mo_0.87_O_2.935_ nanowires were prepared. Furthermore, the diffraction peaks were sharp and intense, showing their high degree of crystallinity.

**Figure 2:**
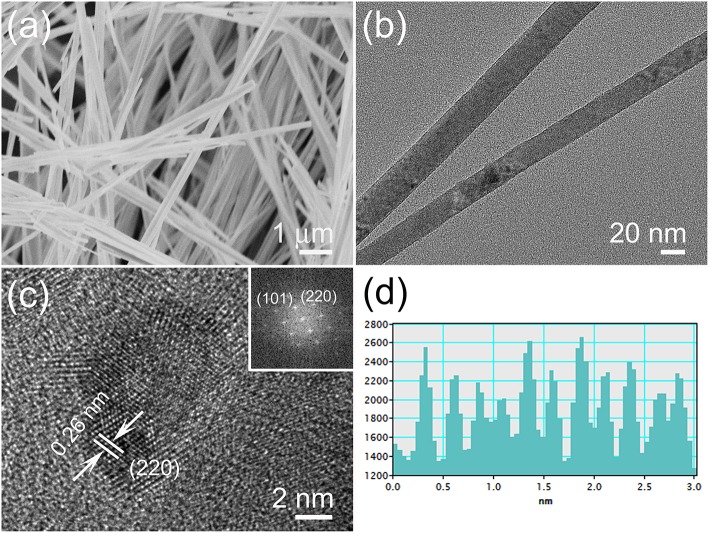
**(a)** SEM, **(b)** TEM, and **(c)** HR-TEM images of the one-dimensional V_0.13_Mo_0.87_O_2.935_ nanowires; the illustration shows the FFT pattern and **(d)** the corresponding lattice spacing obtained from **(c)**.

The SEM image in Figure 2a depicts the typical morphology of the one-dimensional V_0.13_Mo_0.87_O_2.935_ nanowires, which consists of a number of uniform nanowires with an edge length of more than 10 μm. For more detail, the samples were examined by TEM as indicated in Figure 2b in that the diameters of the nanowires are 20–30 nm with uniform nanostructures. The HR-TEM image is indicated in Figure 2c; those one-dimensional V_0.13_Mo_0.87_O_2.935_ nanowires have a similar crystal structure and no amorphous phase on the surface. It could be deduced from the lattice fringes that the lattice spacing is 0.26 nm, agreeing to the (220) plane of one-dimensional V_0.13_Mo_0.87_O_2.935_ nanowires. In further studying the details, the brighter spots in the FFT pattern (illustration in Figure 2c) pointed out an excellent crystal. Besides, Figure 2d confirmed that the lattice spacing of 0.26 nm in Figure 2c belongs to the (220) plane. These results closely matched the data obtained from the XRD analysis, further confirming the crystal structure of V_0.13_Mo_0.87_O_2.935_ nanowires.

**Figure 3:**
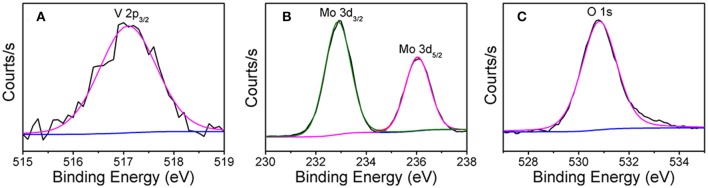
XPS spectra of **(A)** V 2p, **(B)** Mo 3d, and **(C)** O 1s electrons in V_0.13_Mo_0.87_O_2.935_ nanowires.

The X-ray photoelectron spectroscopy (XPS) shows that the one-dimensional V_0.13_Mo_0.87_O_2.935_ nanowires are composed of three elements: V, Mo, and O (Figure S1 of the Supporting Information). The XPS peak of V 2p in Figure 3A was determined to be a peak of V 2p_3/2_ of 517.1 eV, and the V 2p_1/2_ peak of V^5+^ was not included because the low mole percentage of vanadium in the compound was the smallest (Geert et al., 2004; Liu X. et al., 2018). Figure 3B shows the Mo 3d spectrum composed of two peaks, the Mo 3d_3/2_ from the peak at 236.0 eV indicates Mo^6+^, and another peak at 232.9 eV could be due to the superposition of Mo 3d_5/2_ and Mo 3d_3/2_, which indicates Mo^6+^ and Mo^5+^ (Bica de Moraes et al., 2004). Meanwhile, in Figure 3C, the XPS peak of the O 1s was observed at 530.8 eV. In addition, the existence of Mo^5+^ was ascribed to the oxygen anion vacancy in the framework of the compound structure, so that molybdenum is only coordinated by five oxygen species.

**Figure 4:**
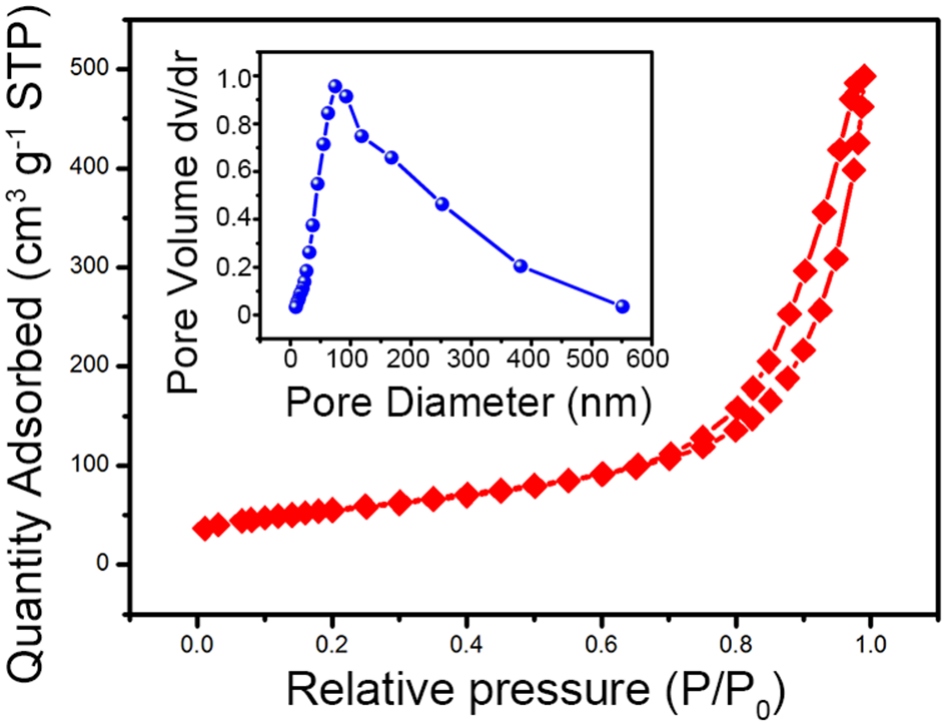
The N_2_ adsorption–desorption isotherm and pore size distributions (illustration) of the one-dimensional V_0.13_Mo_0.87_O_2.935_ nanowires.

The one-dimensional V_0.13_Mo_0.87_O_2.935_ nanowires were further investigated by the N_2_ adsorption–desorption isotherms as indicated in Figure 4. According to IUPAC, the N_2_ adsorption–desorption isotherms of the V_0.13_Mo_0.87_O_2.935_ nanowires are a typical type IV adsorption isotherm with the H3 hysteresis loop, exhibiting a mesoporous structure with slit-shaped pores. The BET-specific surface area and pore diameters (illustration in Figure 4) of the V_0.13_Mo_0.87_O_2.935_ nanowires are about 54.2 m^2^ g^−1^ and 80 nm, respectively, which may be attributed to the assembly of the nanowires in space. This porous structure contributes to the diffusion of electrolyte ions and transport during the charge and discharge process of the SC electrodes (Hou et al., 2018, 2019).

**Figure 5:**
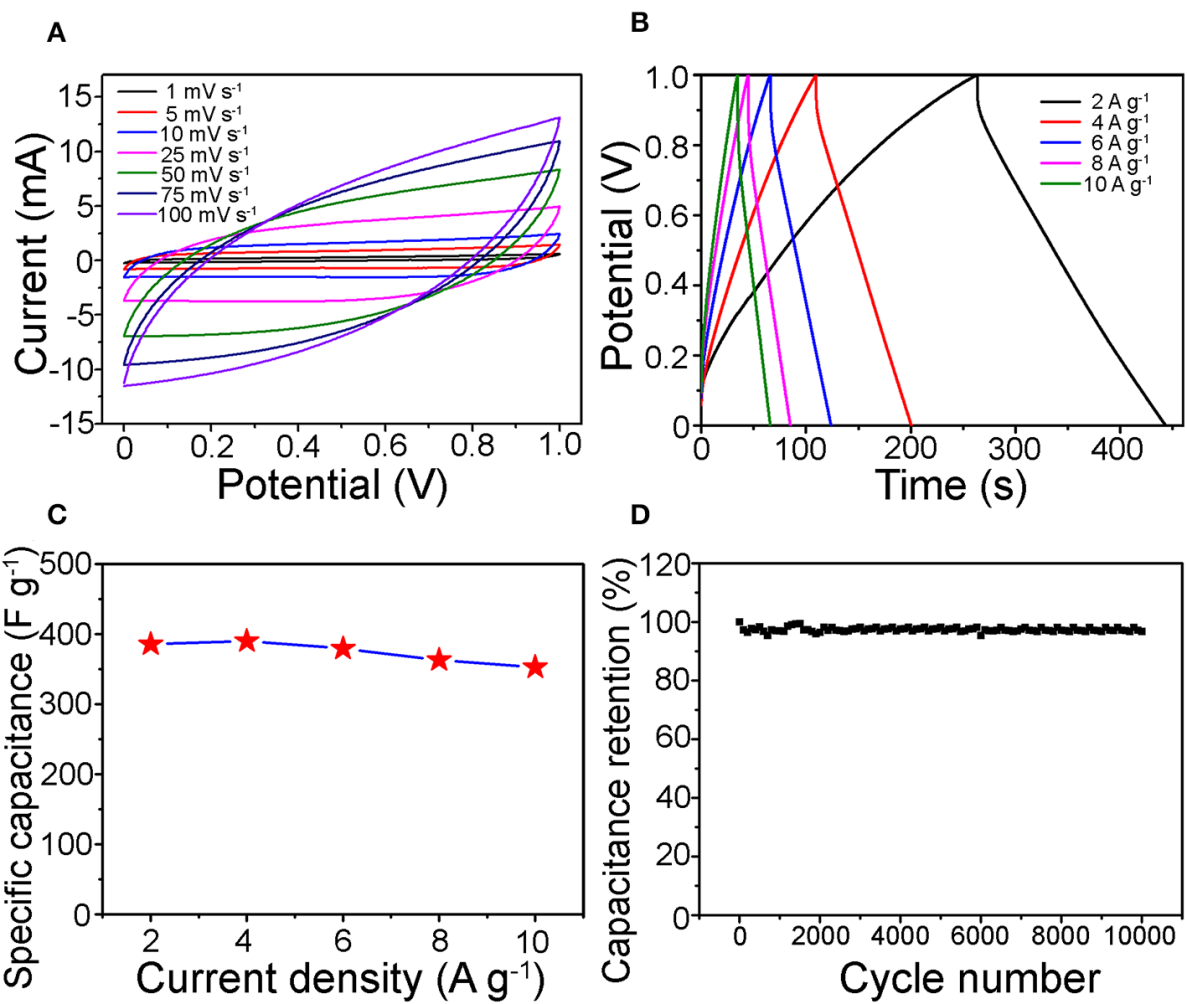
**(A)** CV and **(B)** GCD curves of the one-dimensional V_0.13_Mo_0.87_O_2.935_ nanowire electrodes on different sweeping rates and current densities, respectively. **(C)** Rate performance and **(D)** cycle stability of the V_0.13_Mo_0.87_O_2.935_ nanowire electrodes, in 1 M Na_2_SO_4_ electrolyte.

The as-prepared one-dimensional V_0.13_Mo_0.87_O_2.935_ nanowires were applied to SC electrode materials. Figure 5A depicts the CV curves tested in the voltage from 0 to 1.0 V. Approximate rectangle-shaped and symmetrical CV curves were viewed without redox peaks, showing an EDLC-dominated capacitance behavior of the one-dimensional V_0.13_Mo_0.87_O_2.935_ nanowires (Hung et al., 2011; Lokhande et al., 2011; Pujari et al., 2016). Besides, the specific capacitance (Table S1 of the Supporting Information) of one-dimensional V_0.13_Mo_0.87_O_2.935_ nanowires was very high and was 394.6 F g^−1^ at 1 mV s^−1^. Notably, it can be seen that the CV curve mostly remains in an approximately rectangle-like shape with a sweeping rate between 1 and 100 mV s^−1^, which confirmed good electrochemical reversibility and outstanding high-energy storage performance; the CV plot tilt increases with increasing scan rates owing to the fact that the electrons do not migrate from the inside of the material to the surface of the electrode in time. Figure 5B shows the GCD curves of the one-dimensional V_0.13_Mo_0.87_O_2.935_ nanowire electrode at different current densities. It displayed proximate central symmetry voltage profiles, which were consistent compared to the CV results, pointing to the one-dimensional V_0.13_Mo_0.87_O_2.935_ nanowires having an excellent reversibility across the whole potential region. Furthermore, one-dimensional V_0.13_Mo_0.87_O_2.935_ nanowire electrodes presented high specific capacitances from 385.2 to 352.5 F g^−1^ while discharge current density was enhanced to 2, 4, 6, 8, and 10 A g^−1^ (Table S2 of the Supporting Information). Compared with other binary metal oxide electrodes, one-dimensional V_0.13_Mo_0.87_O_2.935_ nanowire electrodes also indicated a strengthened specific capacitance as reported in the literature, such as CoMoO_4_ (384 F g^−1^) (Li et al., 2018), BiVO_4_ (116.3 F g^−1^) (Patil et al., 2016), and MnMoO_4_ (168.32 F g^−1^) (Veerasubramani et al., 2014).

The specific capacitances of the V_0.13_Mo_0.87_O_2.935_ electrodes with different current densities are indicated in Figure 5C. It maintained a remarkable rate performance of 91.5% from 2 to 10 A g^−1^. This result may be attributed to the active materials to form porous channels through intertwined networks, enabling efficient electrolyte transport and accessibility of active sites (Jiang et al., 2011). Therefore, it is possible to maintain a high specific capacitance even at higher current densities. Figure 5D indicates the long-term cycle stability of the one-dimensional V_0.13_Mo_0.87_O_2.935_ nanowire electrode, which was tested through CV tests repeating 10,000 cycles at 50 mV s^−1^. It can be observed that its specific capacitance retention showed outstanding stability, with the increase in some cycles fluctuating only a little. After 10,000 cycles, the retention rate value was found to be 97.6% of the initial value.

**Figure 6:**
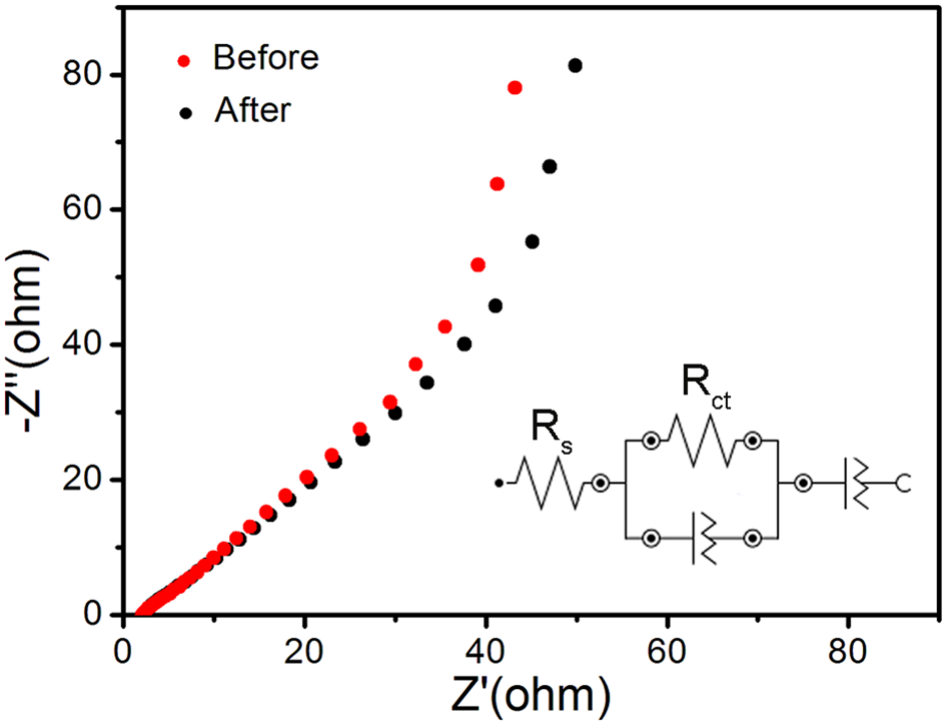
Nyquist plot before and after 10,000 cycles of the one-dimensional V_0.13_Mo_0.87_O_2.935_ nanowire electrodes, with the inset showing the corresponding equivalent circuit.

The V_0.13_Mo_0.87_O_2.935_ electrodes were subjected to electrochemical impedance spectroscopy (EIS) to explore relevant charge transfer resistance. Figure 6 shows the Nyquist plot before and after 10,000 cycles of the one-dimensional V_0.13_Mo_0.87_O_2.935_ nanowire electrodes. The inset shows the corresponding equivalent circuit by its corresponding fitting curve (Figure S2 in Supporting Information), which was fitted by an equivalent circuit consisting of a bulk solution resistance *R*_*s*_, a charge-transfer *R*_*ct*_, and constant phase element (CPE). The *R*_*s*_ values of the one-dimensional V_0.13_Mo_0.87_O_2.935_ nanowire electrode before and after 10,000 cycles are 2.02 and 2.10 Ω, respectively. Also, the value of *R*_*ct*_ was connected with charge transfer after 10,000 cycles and is only slightly higher than before (68.6 vs. 50.1 Ω), manifesting superior conductivity and stability of the one-dimensional V_0.13_Mo_0.87_O_2.935_ nanowire microstructure owing to good ion conductivity of the interface between electrolyte and electrodes.

## Conclusions

In summary, one-dimensional V_0.13_Mo_0.87_O_2.935_ nanowires were synthesized under a facile one-step hydrothermal condition. For application in a SC electrode, it was found to present a high specific capacitance of 394.6 F g^−1^ (1 mV s^−1^). Besides, this electrode showed a perfect rate capability of 91.5% at the current density that was enhanced five times and outstanding long-term cyclic stability (97.6% after 10,000 cycles). This study offers a common preparation method of binary molybdenum–vanadium oxide used in SCs with a superior electrochemical property.

## Data Availability

All datasets generated for this study are included in the manuscript/Supplementary Files.

## Author Contributions

WL conceived and designed the experiments. HJ, WS, and ZWa performed the experiments and analyzed the data. HJ and WS wrote and revised the manuscript. WL, ZWu, XZ, and JB discussed and supervised the whole project. All the authors revised and checked draft.

### Conflict of Interest Statement

The authors declare that the research was conducted in the absence of any commercial or financial relationships that could be construed as a potential conflict of interest.
