# Supplementary material for: Facile Synthesis of Novel V0.13Mo0.87O2.935 Nanowires With High-Rate Supercapacitive Performance
Source: Front Chem. 2019 Sep 4;7:595. doi: 10.3389/fchem.2019.00595 (PMC6737579; doi:10.3389/fchem.2019.00595)
Supplement: Supplementary file 1 [file Table_1.DOC]

Supporting Information

**Facile synthesis of novel** **V_0.13_Mo_0.87_O_2.935_ nanowires with high-rate supercapacitive performance**

Haishun Jiang^1^, Wenjing Sun^1^, Wenyao Li^1^,*, Zhe Wang^1^, Xiying Zhou^1^, * Zexing Wu^2^ and Jinbo Bai^3^, *

^1^School of Material Engineering, Shanghai University of Engineering Science, Shanghai 201620, China.

^2^College of Chemistry and Molecular Engineering, Qingdao University of Science & Technology, 53 Zhengzhou Road, 266042, Qingdao, P. R. China

^3^Laboratoire Mécanique des Sols, Structures et Matériaux, CNRS UMR 8579, Ecole Centrale Supelec, Université Paris Saclay, Grande Voie des Vignes, 92290, Chatenay-Malabry, France.

*Correspondence:

liwenyao314@gmail.com, zhouxiying@sues.edu.cn, jinbo.bai@centralesupelec.fr

**Part I:** **Calculations**

The specific capacitance of the electrode was calculated from the C-V curves according to the following equation^1^:

$$C=\frac{Q}{\Delta V*m}$$

where *C* (F g^-1^) is the specific capacitance, *m* (g) is the mass of the active materials in the electrodes, *Q* (C) is an average charge during the charging and discharging processes, and Δ*V* (V) is the potential window.

The discharge specific capacitance is calculated from the discharge curves using the following formula^1^:

$$C=\frac{I*\Delta t}{m*\Delta V}$$

where *I* (A), Δ*t* (s), *m* (g), and Δ*V* (V) are the discharge current, discharge time consumed in the potential range of ΔV, mass of the active materials, and the potential windows, respectively.

In our work, the mass of active material is ~2.26 mg, and the coating surface area is ~1 cm^2^.

1. J. Yan, E. Khoo, A Sumboja, P. S. Lee. *ACS Nano*, 2010, **4**, 4247-4255.

**Part II: Supplementary Figures and tables**

**
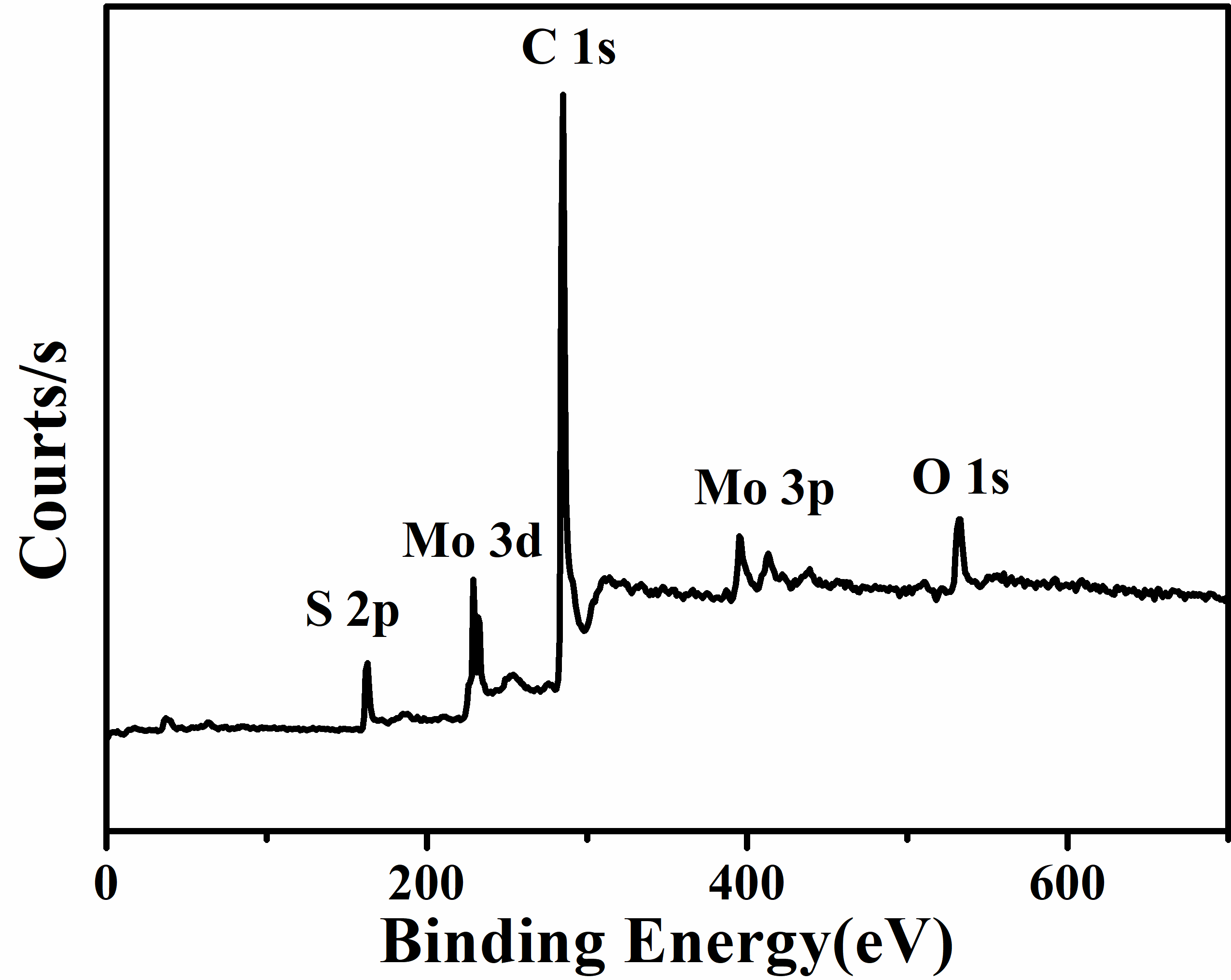
**

Figure S1. XPS complete survey of V_0.13_Mo_0.87_O_2.935_ nanowires


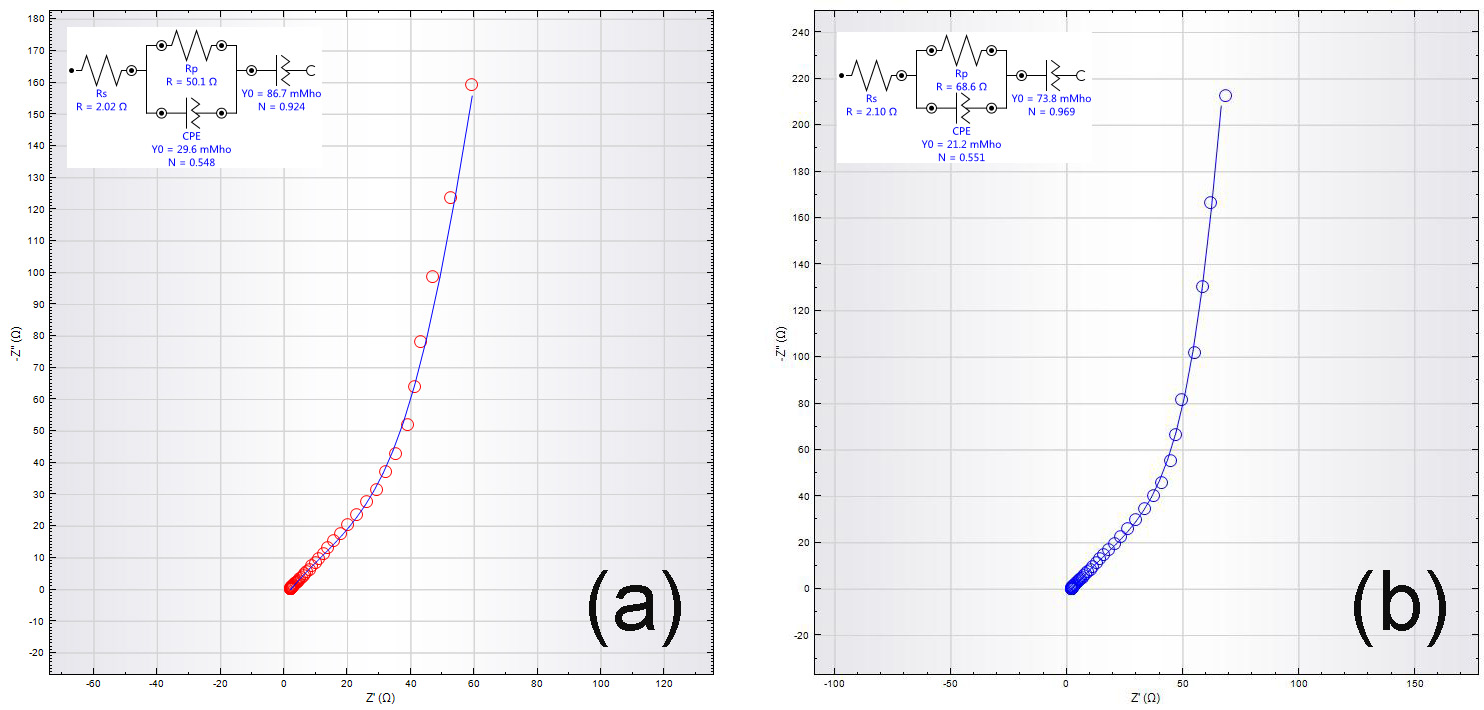


Figure S2. EIS spectra of (a) before and (b) after 10000 cycles of the one-dimensional V_0.13_Mo_0.87_O_2.935_ nanowires electrodes that contain the corresponding fitting curves and equivalent circuit, respectively.

Table S1. Specific capacitance values at different scanning rates

| Scan rate (mV/s) | 1 | 5 | 10 | 25 | 50 | 75 | 100 |
| --- | --- | --- | --- | --- | --- | --- | --- |
| Specific capacitance (F/g) | 394.64 | 307.46 | 290.87 | 249.36 | 197.81 | 164.03 | 140.62 |

Table S2. Specific capacitance values at different current density

| Current density (A/g) | 2 | 3 | 4 | 5 | 6 | 7 | 8 | 9 | 10 |
| --- | --- | --- | --- | --- | --- | --- | --- | --- | --- |
| Specific capacitance (F/g) | 385.21 | 390.64 | 389.84 | 385.88 | 379.19 | 371.86 | 362.97 | 355.77 | 352.51 |
